# Genotyping-by-sequencing derived SNP markers reveal genetic diversity and population structure of *Dactylis glomerata* germplasm

**DOI:** 10.3389/fpls.2025.1530585

**Published:** 2025-02-06

**Authors:** Muhammad Tanveer Altaf, Pablo Federico Cavagnaro, Kağan Kökten, Amjad Ali, Andres Morales, Muhammed Tatar, Mehmet Bedir, Muhammad Azhar Nadeem, Muhammad Aasim, Nafiz Çeliktaş, Sheikh Mansoor, Faheem Shehzad Baloch

**Affiliations:** ^1^ Department of Field Crops, Faculty of Agriculture, Recep Tayyip Erdoğan University, Pazar, Rize, Türkiye; ^2^ Consejo Nacional de Investigaciones Científicas y Técnicas (CONICET), and Instituto Nacional de Tecnología Agropecuaria (INTA) Estación Experimental Agropecuaria Mendoza, Mendoza, Argentina; ^3^ Faculty of Agricultural Sciences and Technologies, Sivas University of Science and Technology, Sivas, Türkiye; ^4^ Universidad Nacional de Cuyo (UNCuyo) Facultad de Ciencias Agrarias, Instituto de Biología Agrícola de Mendoza (IBAM CONICET) Laboratorio de Biología Molecular, Mendoza, Argentina; ^5^ Instituto Nacional de Tecnología Agropecuaria (INTA) Estación Experimental Agropecuaria La Consulta, La Consulta, Argentina; ^6^ Department of Field Crops, Faculty of Agriculture, Hatay Mustafa Kemal University, Hatay, Türkiye; ^7^ Department of Plant Resources and Environment, Jeju National University, Jeju, Republic of Korea; ^8^ Department of Biotechnology, Faculty of Science, Mersin University, Yenişehir, Mersin, Türkiye

**Keywords:** *Dactylis glomerata*, genetic diversity, SNP markers, forage breeding, population structure, GWAS

## Abstract

Orchardgrass (*Dactylis glomerata* L.), a widely cultivated cool-season perennial, is an important forage crop due to its adaptability, high nutritional value, and substantial biomass. Understanding its genetic diversity and population structure is crucial for developing resilient cultivars that can withstand climate change, diseases, and resource limitations. Despite its global significance in fodder production, the genetic potential of many regional accessions remains unexplored, limiting breeding efforts. This study investigates the genetic diversity (GD) and population structure of 91 accessions of *D. glomerata* from Turkey and Iran using genotyping-by-sequencing based single nucleotide polymorphism (SNP) markers. A total of 2913 high-quality SNP markers revealed substantial genetic variability across provinces. Notably, accessions from Erzurum exhibited the highest GD (mean GD: 0.26; He: 0.5328), while provinces such as Bursa and Muğla demonstrated lower GD (mean GD: 0.15; He < 0.22), suggesting potential genetic bottlenecks. Population structure analysis using Bayesian clustering, PCoA and UPGMA dendrograms divided the accessions into three distinct clusters, with cluster membership largely reflecting geographical origins, and dry biomass content. Cluster II revealed higher GD, associated with enhanced biomass production (128 g/plant), the most important agronomic trait in forage species, supporting the notion of heterosis in breeding programs. The majority of the genetic variation (85.8%) was observed within clusters, with minimal differentiation among clusters (FST = 0.007). Genome-wide association studies (GWAS) identified significant marker-trait associations for dry biomass weight, a critical agronomic trait, with markers DArT-100715788, DArT-101043591, and DArT-101171265 and DArT-101090822 located on Chromosomes 1, 6, and 7 respectively. These findings highlight the importance of regional diversity for maintaining adaptive potential in future breeding programs.

## 1 Introduction

*Dactylis glomerata* L., commonly known as Orchardgrass is a member of the gramineous family Poaceae (Mao et al., 2016; Zhang et al., 2022). The genus Dactylis is distinctive and distinctly different from other genera in the Poaceae family, demonstrating significant variance in taxonomic characteristics and flourishing in a wide range of environments (Lumaret, 1997). Despite having just one species, *D. glomerata*, the genus Dactylis has at least eighteen subspecies (Sanada et al., 2010). It is regarded as the fourth most significant wild forage grass globally (Chtourou-Ghorbel et al., 2024). This perennial cool-season grass grows natively in North Africa, West and Central Asia, the Mediterranean basin, and Europe (Stewart and Ellison, 2010; Xie et al., 2014; Lee et al., 2021).

Diploid, tetraploid, and some hexaploid populations of *D. glomerata* occur naturally (Lolicato and Rumball, 1994; Sanada et al., 2010). Most diploid populations have limited distributions and are found in specific regions, collectively representing about 5% of all wild *Dactylis*. In contrast, tetraploids are widespread, occurring continuously throughout temperate regions of Europe, the Middle East, West and Central Asia, and North Africa (Lumaret and Borrill, 1988). Both ploidy types often coexist in certain areas while hexaploids are confined to restricted areas in Libya and Western Egypt (Jones and Borrill, 1962; Last et al., 2013). The species exhibits an infraspecific polyploid complex in grasses, resulting from autopolyploidy due to polysomic inheritance. Polyploid populations possess evolutionary advantages stemming from greater heterozygosity and reduced inbreeding depression, facilitating enhanced colonization and adaption to fluctuating ecological conditions (Soltis and Soltis, 2000; Van de Peer et al., 2021).

This is an important fodder crop due to its favorable palatability and high sugar content for animals, supporting animal feed and enhancing dairy and meat production in temperate climates (Wilkins and Humphreys, 2003; Katoch, 2022). It can withstand shade, barrenness, and drought. Furthermore, the rapid growth of its root system makes it particularly valuable as a cover crop for preventing surface erosion and rehabilitating degraded soils (Costa et al., 2016; Copăcean et al., 2019). Likewise, its deep root system allows it to access water and nutrients, making it drought-tolerant and providing high-quality forage for efficient production (Gaier et al., 2024).

Genetic diversity is a critical resource in any breeding program (Iqbal et al., 2023; Altaf et al., 2024a; Yalinkiliç et al., 2024) aimed at improving traits such as yield, disease resistance, abiotic stress tolerance, and forage quality (Jovovic et al., 2020; Baloch et al., 2024). Genetic variations within populations enable breeders to select desirable traits and develop varieties suited to specific environmental conditions or management practices (Bunjkar et al., 2024; Altaf et al., 2024b). Given the widespread cultivation of *D. glomerata*, understanding the genetic heterogeneity within germplasm collections is essential for breeding programs focused on enhancing adaptability, resilience, and productivity (Zhang et al., 2022). Despite its significance, *D. glomerata* faces substantial challenges from both biotic and abiotic stresses, which are likely to be exacerbated by climate change. This underscores the urgency of developing resilient cultivars to ensure its continued productivity and adaptability (Shahzad et al., 2021). Breeding for disease resistance is particularly important to maintain forage yield and quality (Capstaff and Miller, 2018). Traditional phenotypic selection is slow and resource-intensive, whereas modern marker-assisted selection (MAS) provides targeted approaches that rely on understanding genetic diversity and key traits (Nadeem et al., 2018). Different types of molecular markers (SSR, simple sequence repeat; AFLP, amplified fragment length polymorphism; DArT, diversity arrays technology; ISSR, inter simple sequence repeats; and RAPD, randomly amplified polymorphic DNA) have been developed and successfully applied in the marker-assisted breeding program of various crops (Nadeem et al., 2018). However, molecular markers that are resolved through gel-based electrophoresis face limitations such as low reliability, limited genome coverage, labor-intensive processes, and high costs or sequence needs (Jaccoud et al., 2001). These drawbacks affect their use in many crops, particularly ‘orphan’ crops and polyploid species (Sánchez-Sevilla et al., 2015).

New markers developed through Next Generation Sequencing platforms (NGS) are the current prime alternative for molecular studies since they cover a vast proportion of the genome (Kilian et al., 2012). Genotyping-by-sequencing (GBS) is a prevalent NGS technique designed for the concurrent identification of novel markers and genotyping of specific germplasm (Elshire et al., 2011; Rayaprolu et al., 2022). This technology is high-throughput and cost-effective, utilized across multiple crops for diverse applications (Elshire et al., 2011; Poland and Rife, 2012; Huang et al., 2014; Alipour et al., 2017; Geleta et al., 2020; Baloch and Nadeem, 2022). The predominant DNA markers produced with the GBS approach are single nucleotide polymorphisms (SNPs). These markers signify the predominant sequence-based variations within crop genomes, rendering them exceptionally appropriate for the examination of genetic variability, marker-trait association, population structure, mapping quantitative trait loci (QTL), genomic selection, map-based cloning, and various plant breeding applications (Batley and Edwards, 2007; Hiremath et al., 2012; Tsehay et al., 2020; Gardoce et al., 2023; Altaf et al., 2024c).

Advancements in sequencing technologies have significantly advanced statistical genetic methods, with genome-wide association studies (GWAS), enabling the identification of genes or alleles linked to specific traits (Sahito et al., 2024; Ahmed et al., 2024). GWAS utilizes next-generation sequencing (NGS) data, to examine thousands of genetic variants, most commonly single nucleotide polymorphisms (SNPs), across diverse genomes to identify those statistically associated with specific traits. This methodology surpasses the limitations of traditional gene and quantitative trait loci (QTL) mapping, which rely on biparental crosses and are constrained by limited allelic diversity and genomic resolution (Borevitz and Nordborg, 2003). Unlike traditional methods, GWAS utilizes phenotypically characterized germplasm collections, eliminating the need for structured populations (e.g., F2 or F3 progenies), while providing greater allelic diversity and higher mapping resolution, sometimes to the gene level (Tibbs Cortes et al., 2021). This approach has been instrumental in identifying QTL that explains substantial phenotypic variation in a variety of plant traits. Once a phenotype-marker association is identified, downstream analyses of candidate genes in nearby genomic regions can yield valuable insights into the underlying biology of the trait. It has been successfully employed in various plants, including *Arabidopsis*, rice, soybean, wheat, cotton, sorghum, common bean, and Orchardgrass, to study traits ranging from yield to abiotic and biotic stress tolerance (Liu and Yan, 2019; Sukumaran and Yu, 2014; Varshney et al., 2012; Xu et al., 2024; Altaf et al., 2024d).

Studies assessing genetic diversity in *D. glomerata* using SSR, restriction fragment length polymorphism (RFLP), and start codon targeted (SCoT) markers are documented (Madesis et al., 2014; Jiang et al., 2015; Costa et al., 2016), but the use of SNP markers for this purpose remains limited. No studies have yet utilized the GBS approach for SNP-based genetic diversity analysis in this crop. The present study aimed to evaluate the genetic diversity and population structure of 91 accessions of *D. glomerata* using GBS-based SNP markers.

## 2 Materials and methods

### 2.1 Plant materials and field trail

**Figure 1:**
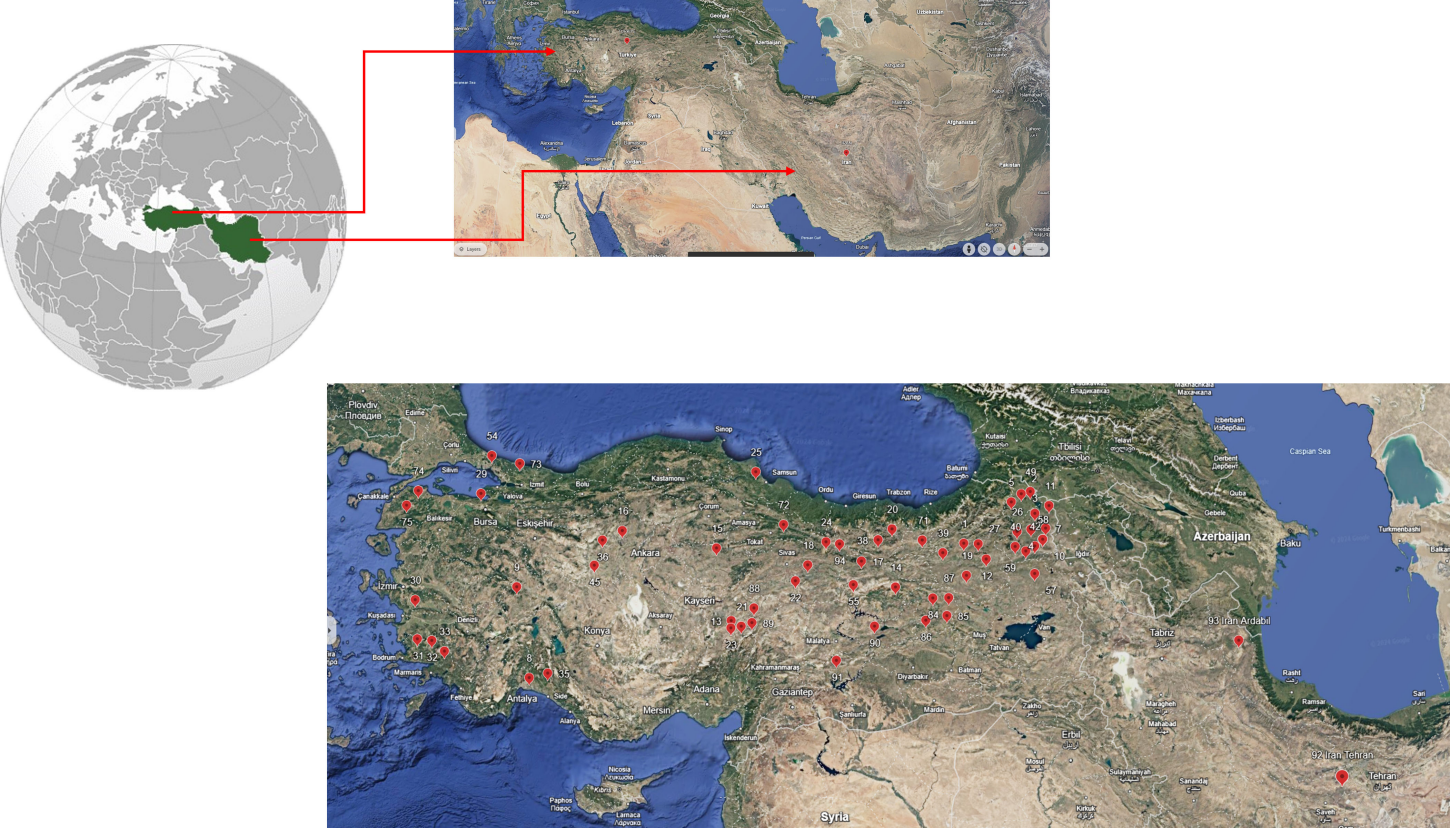
Collection points of the studied *Dactylis glomerata* L germplasm.

A total of 91 *Dactilis glomerata* accessions were used as plant material in this study (**Supplementary Table S1**). The analyzed accessions belong to Turkey and Iran (**Figure 1**) and they were provided by the United States Department of Agriculture (USDA). The field trial was conducted at the experimental field of Sivas University of Science and Technology (Sivas, Turkey) during the growing season from April 2023 to June 2024, following an augmented experimental design. The experimental layout comprised four blocks with a row spacing of 70 cm, inter-row spacing of 70 cm with five plants per row. Sowing was performed in the last week of April 2023, aligning with the regional climatic conditions. As a basal fertilizer, DAP was applied at a rate of 4 kg/da of pure nitrogen (N) and 10 kg/da of pure phosphorus (P_2_O_5_). When the plants reached a height of approximately 25-30 cm, an additional application of ammonium nitrate fertilizer was made at a rate of 6 kg of pure N per decare. The final harvest was done in June 2024. Agromorphological traits analyzed included main stem length (cm), number of tillers per plant, fresh biomass weight (g/plant), and dry biomass weight (g/plant). Fresh weight was measured at the time of harvesting while dry biomass weight was assessed after drying samples in an oven at 65°C for 48 hours until a constant weight was achieved. The data on agromorphological traits measured is provided in **Supplementary Table S2**.

### 2.2 DNA extraction

For genomic DNA isolation, healthy young leaves from each accession were selected and harvested (a single plant per accession), from which total DNA was isolated following the CTAB protocol (Doyle and Doyle, 1990) and with a Diversity Arrays Technology-recommended methodology (available at https://www.diversityarrays.com/orderinstructions/plant-dnaextraction-protocol-for-dart/). The DNA quantity was first checked through 0.8% agarose gel electrophoresis and further quantification was performed with a Nanodrop spectrophotometer (DeNovix DS-11 FX, USA) (Baloch et al., 2024) Samples were diluted to a final concentration of 50 ng μl^-1^ for ‘GBS’ library preparation and sequencing. The DNA samples were processed for DArTseq analysis using a GBS platform at Diversity Array Technology Pty, Ltd, Australia (http://www.diversityarrays.com/).

### 2.3 GBS analysis for SNP markers

DArTseq technology integrates a complexity reduction technique with NGS platforms (Kilian et al., 2012; Elshire et al., 2011), enabling the targeted selection of genome regions linked to key plant traits (Li et al., 2015). For optimizing DArTseq in *D. glomerata*, genome fraction selection and representation size were adjusted accordingly. The complexity reduction employed PstI-MseI enzymes, and DNA samples underwent digestion/ligation reactions as described by Kilian et al. (2012). Mixed fragments (PstI–MseI) were amplified using 30 PCR cycles with the following thermal profile: (I) 94°C for 1 min, (II) 94°C for 20 s, (III) ramp 2.4°C/s to 58°C, (IV) 58°C for 30 s, (V) ramp 2.4°C/s to 72°C, (VI) 72°C for 45 s, (VII) repeat steps 2–6 for 29 cycles, (VIII) 72°C for 7 min, (IX) hold at 10°C (Kilian et al., 2012). Amplified products from each sample in a 96-well microtiter plate were pooled equimolarly, then processed on a c-Bot (Illumina) for bridge PCR, followed by sequencing on an Illumina HiSeq2000 platform. Sequencing involved 77 single-read cycles. Data processing was conducted via proprietary DArT analytical pipelines (Li et al., 2015), starting with filtering fastq files to eliminate low-quality sequences, applying stringent quality criteria to the barcode region for accurate sample assignment during barcode splitting. Approximately 4,000,000 sequences per sample were analyzed for marker calling. Identical sequences were condensed into “fastqcall files” for secondary analysis in DArT PL’s proprietary pipeline, which included SNP and SilicoDArT (presence/absence of restriction fragments) marker identification using DArTsoft14 software.

### 2.4 Statistical analyses

Raw data were loaded and filtered in R (R Core Team, 2021) version 4.2 using the dartR package v2 (Gruber et al., 2022; Mijangos et al., 2022) with the following criteria. All SNPs that had > 10% missing data were removed, as well as markers missing in all individuals of at least one population, considering as populations the geographical origin of the accessions. Markers exhibiting a repeatability score (RepAvg) of less than 80% were eliminated, along with those derived from an identical DNA fragment, as they were deemed redundant (non-informative). SNPs with a minor allele frequency (MAF) lower than 5% were also discarded. Subsequently, missing data were imputed using method = “neighbour”. The resulting SNPs data were used for genetic analyses in the *Dactilis glomerata* germplasm collection.

Simple agglomerative hierarchical clustering was performed using poppr R package (Kamvar et al., 2024, 2014). Pair-wise genetic dissimilarity (GDi) values using Hamming distance were calculated among the accessions with the ‘bitwise.dist’ function. Following the calculation of GDi values a distance matrix was generated and used to construct dendrograms using the Unweighted Paired Group Method with Arithmetic means (UPGMA) with ‘aboot’ and visualized using the package ‘ggtree’ (Yu et al., 2017; Yu et al., 2021). Principal coordinate analysis (PCoA) was performed using ‘gl.pcoa’, a wrapper function implemented in dartR v2, and the first two principal coordinates were plotted. The genetic structure of the populations was assessed with Bayesian clustering algorithms of the fastSTRUCTURE software (Raj et al., 2014), an implementation of STRUCTURE (Pritchard et al., 2000) specifically made to handle genomic SNP matrix data. Distruct barplots were constructed in R using the package ‘pophelper’ (Francis, 2024). Selection of the optimum number of populations (K) was done using the *post hoc* methods proposed previously (Evanno et al., 2005), by running fastSTRUCTURE with 100 replicates of K ranging from 1 to 15, and the most parsimonious model was selected based on their mean likelihood and their delta K. Analysis of molecular variance were performed using pegas AMOVA as implemented in dartR (Mijangos et al., 2022) using i) geographical origin and ii) clusters inferred from UPGMA tree as subpopulations. General genetic statistics including, minor allele frequency; observed heterozygosity; expected heterozygosity, inbreeding coefficient were independently calculated for populations (provinces) and UPGMA genetic clusters using the ‘popgen’ function implemented in the snpReady package (Granato and Fritsche-Neto, 2018; Granato et al., 2018).

### 2.5 Genome-Wide Association Mapping for dry biomass weight

To investigate marker-trait associations (MTAs), a mixed linear model (MLM; Q + K) approach was implemented using TASSEL 5.0.5 (Bradbury et al., 2007). We had genotypic data provided by Diversity Arrays Technology with no chromosomes and known chromosomal positions of each marker. Therefore, we generated puso-chromosomes in order to develop the pseudo-Manhattan plot for the visualization of linked DArT loci associated with the studied trait. The population structure and familial relationships were accounted for by incorporating Q-metrics (Q) and kinship (K) into the association analysis, following the methodology suggested by Nadeem et al. (2020). The kinship matrix was estimated using the scaled identity by descent method available in TASSEL 5.0.5 (Bradbury et al., 2007). In the association analysis results, the p-value indicates the statistical significance of the relationship between a marker and the associated trait, while R² represents the proportion of phenotypic variation explained by the significant marker (Jin et al., 2011). FDR threshold cut-off = 0.0001 was used to identify a statistically significant marker-trait association. We decided to report only those SNPs with -log10(p-value) > 3.8 for declaring significant-marker trait associations. This more stringent threshold was chosen to reduce the risk of false positives and improve the reliability of the identified associations (Borrego-Benjumea et al., 2021). Such conservative thresholds are consistent with best practices in GWAS to balance type I and type II errors when analyzing complex traits. A pseudo-Manhattan plot was generated using the qqman package in R 4.0.0 (Turner, 2014) to visualize the results.

## 3 Results

The GBS analysis yielded, after data filtering, a total of 2, 913 high-quality SNPs across 91 *D. glomerata* accessions. The genetic diversity indices for each geographical origin were measured based on provinces (**Table 1**). There was a noticeable range in mean genetic diversity (GD), polymorphism information index (PIC), observed heterozygosity (Ho), and expected heterozygosity (He) values across provinces. Accessions from Erzurum exhibited the highest GD, with a mean of 0.26 and He of 0.5328, and PIC value (0.21), indicating significant genetic variability within this province. Similarly, accessions from Çanakkale and Sivas also showed high diversity levels (**Supplementary Table S2**).

**Table 1:** Genetic diversity statistics by geographical origin of the accessions.

Geographical origin	Number of accessions (%)	mean GD	mean PIC	mean MAF	mean Ho	mean He	mean Fi
Afyon	1 (1.1%)	0.2	0.16	0.14	0.24	0.2904	-0.21
Agri	1 (1.1%)	0.19	0.16	0.13	0.22	0.2552	-0.16
Ankara	3 (3.2%)	0.19	0.15	0.13	0.22	0.253	-0.15
Antalya	2 (2.1%)	0.17	0.14	0.12	0.19	0.2109	-0.11
Ardahan	3 (3.2%)	0.19	0.16	0.13	0.22	0.2574	-0.17
Aydin	1 (1.1%)	0.18	0.14	0.12	0.18	0.1926	-0.07
Ayfam	1 (1.1%)	0.19	0.16	0.14	0.23	0.2691	-0.17
Bayburt	1 (1.1%)	0.18	0.15	0.13	0.22	0.2684	-0.22
Bingöl	4 (4.3%)	0.18	0.15	0.13	0.23	0.299	-0.3
Bursa	1 (1.1%)	0.15	0.12	0.11	0.16	0.1696	-0.06
Çanakkale	2 (2.1%)	0.21	0.17	0.16	0.28	0.3696	-0.32
Elazgi	1 (1.1%)	0.18	0.15	0.13	0.21	0.2373	-0.13
Erzican	1 (1.1%)	0.2	0.17	0.15	0.26	0.3328	-0.28
Erzurum	6 (6.4%)	0.26	0.21	0.19	0.37	0.5328	-0.44
Gümüşhane	2 (2.1%)	0.18	0.15	0.13	0.19	0.2014	-0.06
Erdebil (Iran)	1 (1.1%)	0.16	0.13	0.12	0.2	0.238	-0.19
Istanbul	2 (2.1%)	0.17	0.14	0.12	0.2	0.24	-0.2
Kars	11 (11.7%)	0.16	0.13	0.12	0.17	0.1751	-0.03
Kayseri	5 (5.3%)	0.16	0.13	0.11	0.19	0.228	-0.2
Malatya	1 (1.1%)	0.19	0.15	0.14	0.22	0.253	-0.15
Mugla	3 (3.2%)	0.15	0.12	0.11	0.18	0.216	-0.2
Samsun	1 (1.1%)	0.17	0.14	0.13	0.2	0.226	-0.13
Sivas	4 (4.3%)	0.21	0.17	0.16	0.3	0.417	-0.39
Taya	1 (1.1%)	0.21	0.17	0.15	0.27	0.3483	-0.29
Tunceli	1 (1.1%)	0.2	0.17	0.15	0.27	0.3591	-0.33
Yozgat	1 (1.1%)	0.15	0.13	0.11	0.17	0.1904	-0.12
Turkey	30 (31.9%)	0.2	0.16	0.14	0.26	0.3406	-0.31

GD, Genetic diversity; PIC, Polymorphic index content; MAF, Minor allele frequency; Ho, Observed heterozygosity; He, Expected heterozygosity; Fi, Inbreeding coefficient.

The analysis of molecular variance (AMOVA) on provincial level revealed that the majority of the genetic variation (95.4%) was attributed to differences among populations based on provincial divisions. In contrast, a smaller proportion of the variation (4.6%) occurred among individual accessions within populations (**Table 2**). The overall pair-wise genetic differentiation (FST) was 0.143 (**Table 2**).

**Table 2:** The analysis of molecular variance and pair-wise genetic differentiation based on provinces.

Source of variation	Variation (%)
Among populations	95.4%
Among accessions	4.6%
Overall FST	0.143

**Figure 2:**
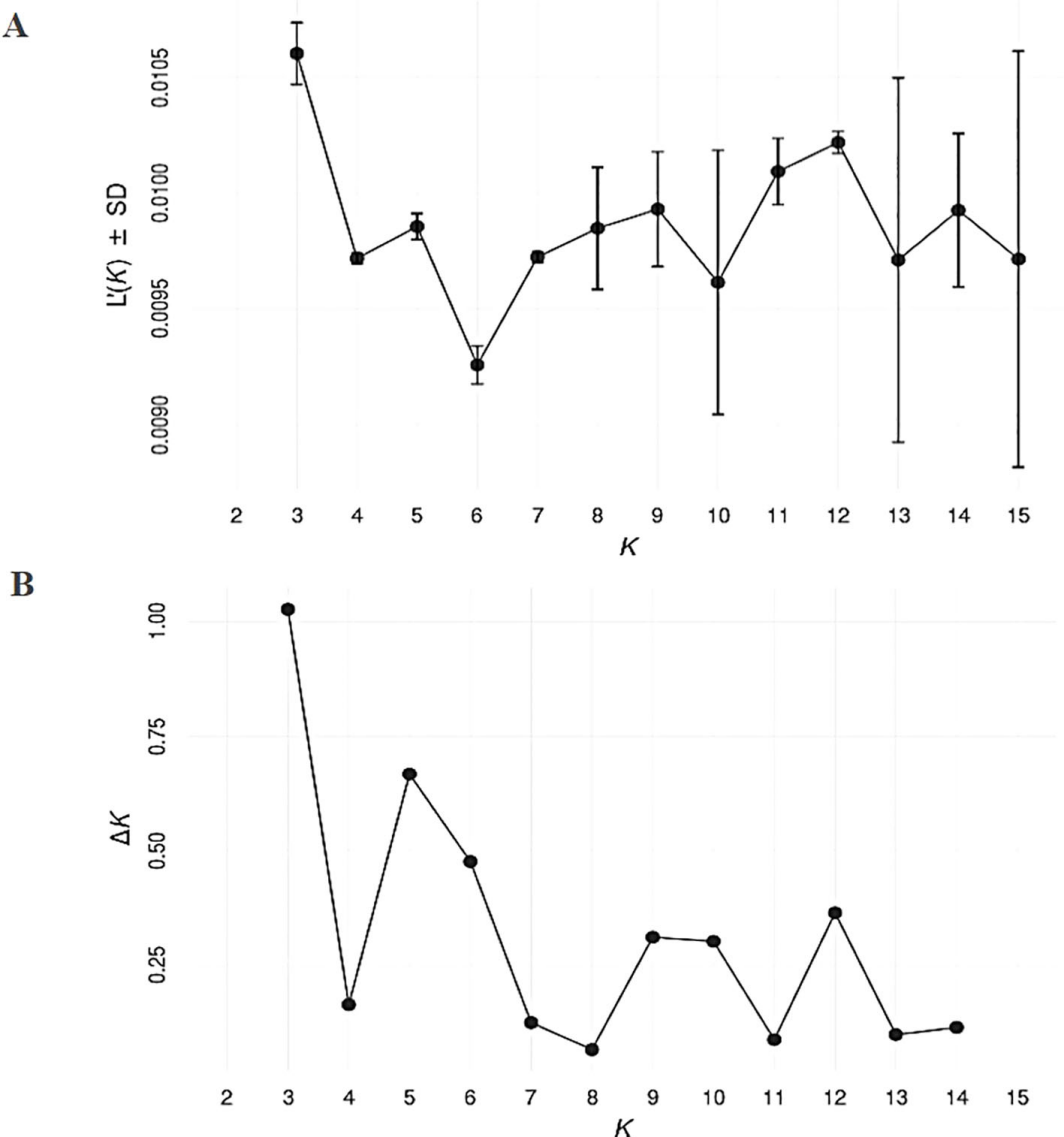
Analysis of the optimum number of populations (K) for estimating genetic structure with SNP data. The *post hoc* method proposed by Evanno et al. (2005) was used with 100 replicates of K ranging from 1 to 12, considering their mean likelihood **(A)** and delta K **(B)** as criteria for selection of the most parsimonious model.

**Figure 3:**
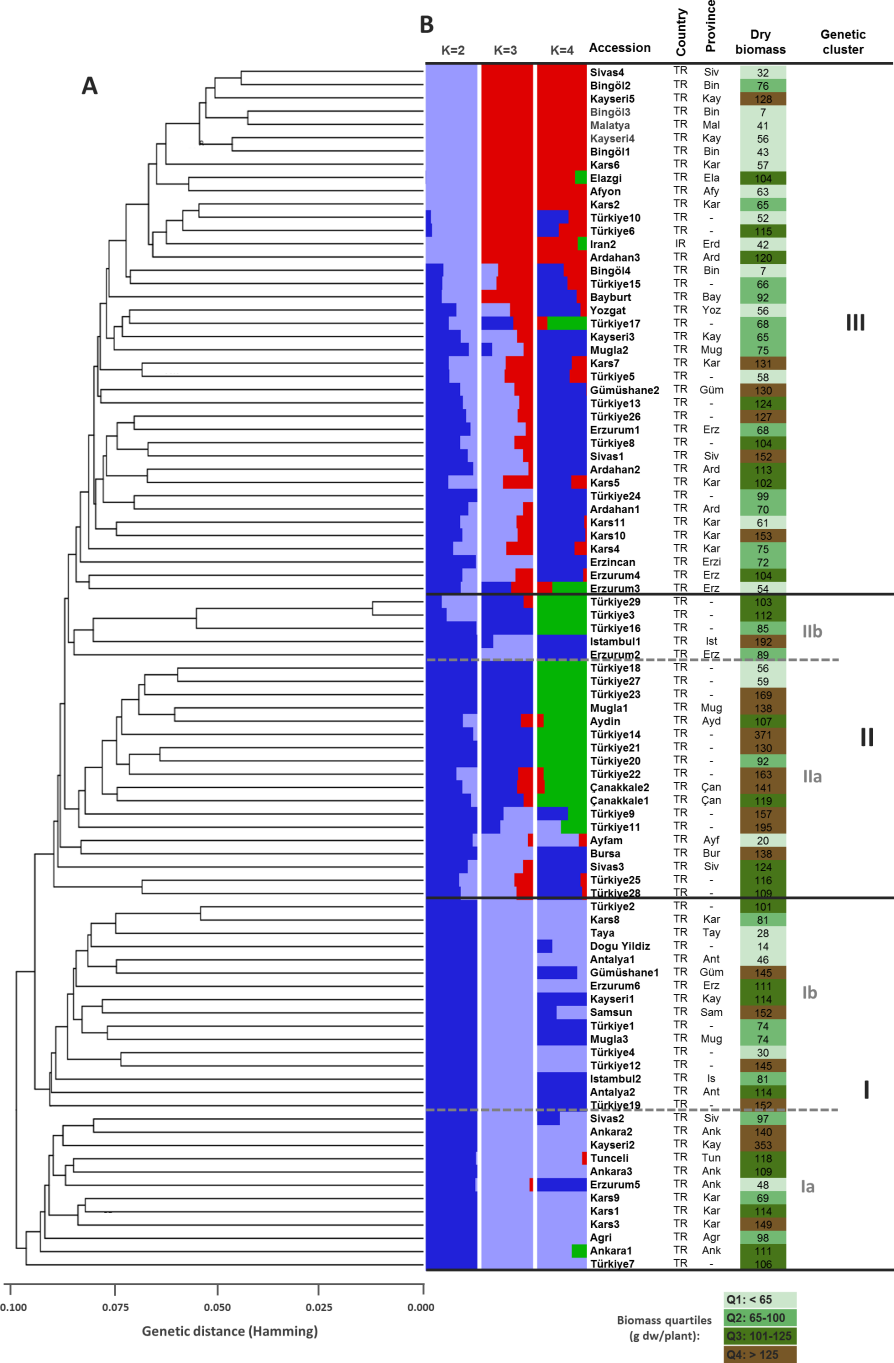
Genetic relationships and population structure for 91 D. glomerata accessions based on 2937 SNP markers. **(A)** UPGMA dendrogram based Hamming genetic distance (GD). Major clusters (I, II, and III) and sub-clusters within clusters I (Ia, Ib) and II (IIa, IIb) are delimited by bold black and gray dashed lines. **(B)** Genetic structure of the accessions considering different optimal number of populations (i.e., K=2, K=3, and K=4). Each accession is represented by a horizontal bar partitioned into two (K=2), three (K=3), or four-colored segments (K=4), indicating their relative membership to the considered clusters. ISO country codes are as follows: TR: Turkey; IR: Iran. Abbreviations of province names are as follows: Afy, Afyon; Ağr, Ağrı; Ank, Ankara; Ant, Antalya; Ard, Ardahan; Ayd, Aydin; Bay, Bayburt; Bin, Bingöl; Bur, Bursa; Çan, Çanakkale; Ela, Elazığ; Erd, Erdebil (Iran); Erzi, Erzincan; Erz, Erzurum; Güm, Gümüşhane; Ist, Istambul; Kar, Kars; Kay, Kayseri; Mal, Malatya; Muğ, Muğla; Sam, Samsun; Siv, Sivas; Tun, Tunceli; Yoz, Yozgat. Dry biomass values are expressed in grams of dry weight per plant (g dw/plant) and quartiles for this trait are color-coded.

SNP markers data was used for genetic structure analysis, using the Bayesian clustering model implemented in the STRUCTURE software. Selection of the optimum number of populations (K) was done using the *post hoc* methods, based on their mean likelihood and their delta K (**Figure 2**). The structure divided the studied germplasm into 3 populations (**Figure 3A**). The pair-wise genetic dissimilarity values among 91 *D. glomerata* accessions, based on 2,937 SNP markers, were calculated using Hamming distance to generate a genetic distance matrix. This matrix was used to construct a dendrogram with the UPGMA. The UPGMA analysis divided the studied accessions into three main clusters (I, II, and III), with further sub-structuring observed within clusters I and II, as indicated by bold black and gray dashed lines in **Figure 3B**. Cluster I comprised 32 accessions (34.04%), which were further divided into two sub-clusters: Ib and Ia. Sub-cluster Ib contained 20 accessions, including those from various provinces such as Antalya, Kayseri, Muğla, and Erzurum. Specifically, these accessions were Türkiye1, Antalya1, Kayseri1, Gümüşhane1, Samsun1, Türkiye2, Muğla3, Antalya2, Türkiye4, Kars8, Türkiye12, Erzurum6, Türkiye19, Tokat1, İstanbul2, Türkiye31, Türkiye32, Türkiye33, Malatya1, and Tahran1. Sub-cluster Ia consisted of 10 accessions from Kars, Tunceli, Ankara, Sivas, Erzurum, and Ağrı, including Kars1, Kars3, Tunceli1, Ankara1, Kayseri2, Sivas2, Ankara2, Erzurum5, Türkiye7, and Ankara3.

Cluster II contained a total of 22 accessions (23.40%), which were also divided into two sub-clusters, IIb and IIa. Sub-cluster IIb consisted of 5 accessions, including Erzurum2, Türkiye3, İstanbul1, Türkiye16, and Türkiye29. The remaining 17 accessions formed sub-cluster IIa, encompassing accessions from Sivas, Bursa, Aydın, Muğla, and other provinces, such as Sivas3, Bursa1, Aydın1, Muğla1, Türkiye9, Türkiye11, Türkiye14, Türkiye18, Türkiye20, Türkiye21, Türkiye22, Türkiye25, Çanakkale1, Çanakkale2, Türkiye27, Türkiye28, and Ayfam (**Figure 3B**).

**Figure 4:**
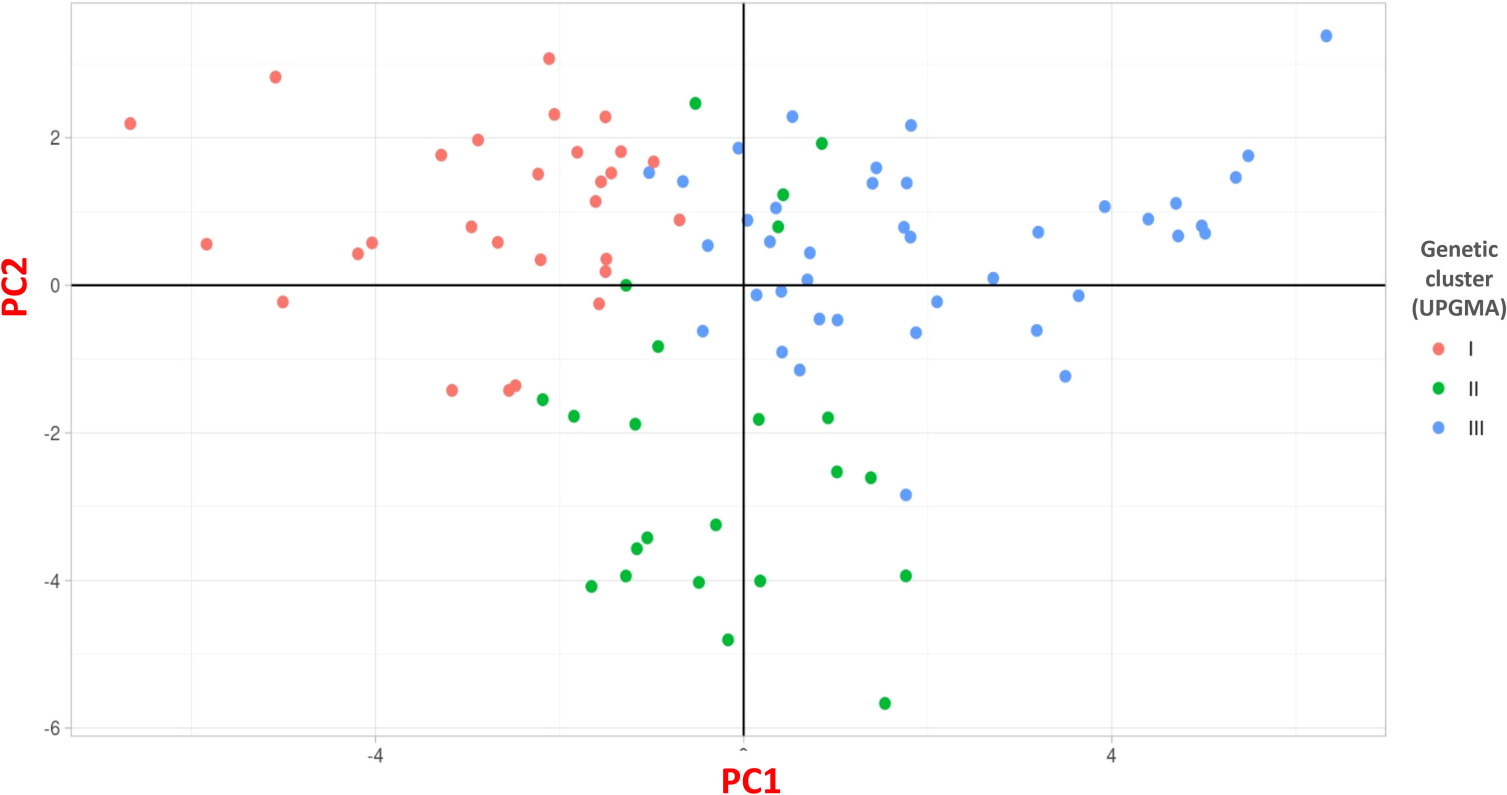
Principal coordinate analysis (PCoA) of the 91 *D. glomerata* accessions using SNP markers.

**Figure 5:**
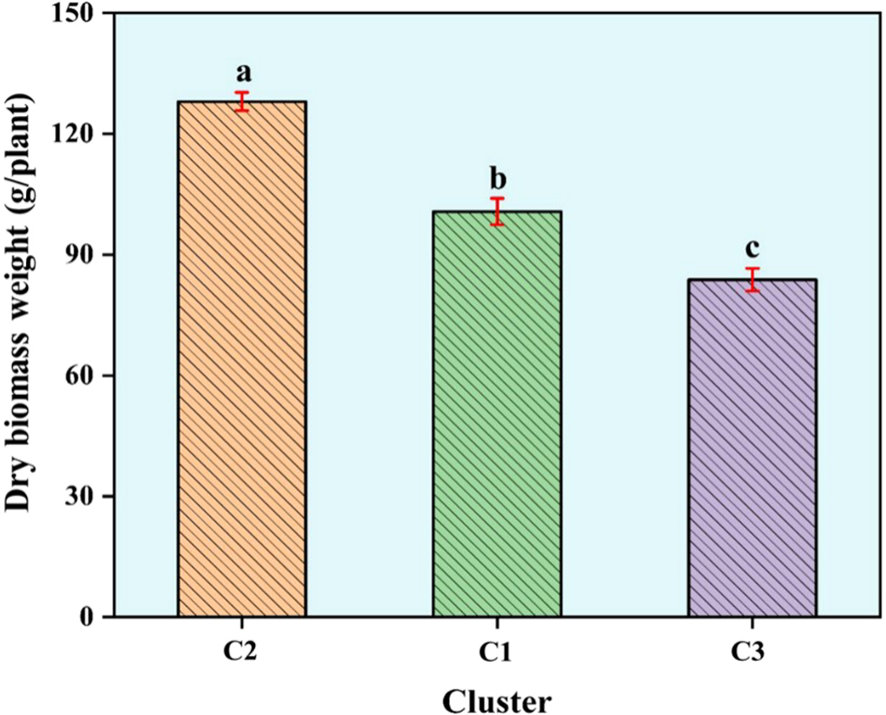
Histogram of dry biomass weight based on UPGMA clustering. Bars represent mean values for each cluster ± standard error. Mean values with different letters are statistically significant using least significant difference (LSD) test at p<0.05.

Cluster III was the largest, containing 40 accessions (42.55%). This cluster included accessions from diverse locations such as Erzurum, Ardahan, Kars, Yozgat, and Bingöl, with notable entries being Erzurum1, Ardahan1, Kars2, Ardahan2, Afyon1, Kars4, Kars5, Yozgat1, Erzincan3, Sivas1, Erzurum3, Kayseri3, Kars6, Erzurum4, Muğla2, Gümüşhane2, Kars7, Türkiye5, Türkiye6, Türkiye8, Ardahan3, Kars10, Kars11, Türkiye15, Türkiye10, Türkiye13, Türkiye23, Türkiye24, Bayburt1, Türkiye26, Türkiye17, Bingöl1, Bingöl2, Bingöl3, Bingöl4, Kayseri4, Kayseri5, Elazığ1, Erdebil1, and Sivas4 (**Figure 3B**). The Principal Coordinates Analysis (PCoA) categorized the germplasm into three distinct groups, consistent with the UPGMA clustering. Accessions within these groups were visually represented using three different colors: red for Group 1, green for Group 2, and blue for Group 3, highlighting their genetic differentiation (**Figure 4**). We collected comprehensive phenotypic data for the germplasm, with a specific focus on dry biomass weight (DBW). Through UPGMA clustering analysis, we found that accessions with similar average DBW values were closely grouped, suggesting a relationship between genetic clustering patterns and phenotypic similarity. To investigate this relationship further, we constructed histograms showing the mean DBW of all the accessions within each UPGMA-based cluster. The results indicated that Cluster II had the highest mean DBW, being significantly greater (p<0.05) than the mean DBW of Clusters I and III, whereas Cluster III exhibited the lowest mean DBW (**Figure 5**).

The diversity indices were also calculated based on clustering and **Table 3** summarizes the genetic diversity statistics for the three major clusters identified. Cluster I consisted of 28 accessions (29.8% of the total) and exhibited a mean genetic diversity (GD) of 0.22, with a polymorphic information content (PIC) of 0.19 and a minor allele frequency (MAF) of 0.14. Observed heterozygosity (Ho) was 0.23, and expected heterozygosity (He) was 0.2369. The inbreeding coefficient (Fi) was slightly negative (-0.03). Cluster II comprised 23 accessions (24.5%), and genetic diversity metrics with a mean GD of 0.22, PIC of 0.18, and MAF of 0.14. The Ho was 0.23, and He was 0.2461, slightly higher than in Cluster I. The negative inbreeding coefficient (Fi = -0.07). Cluster III was the largest cluster, including 40 accessions (42.6%). The genetic diversity metrics were slightly lower than those of the other clusters, with a mean GD of 0.21, PIC of 0.18, and MAF of 0.14. The observed and expected heterozygosity were 0.21 and 0.2121, respectively, with a near-zero inbreeding coefficient (Fi = -0.01. Furthermore, the AMOVA was also calculated on cluster base and revealed that the majority of genetic variation (85.8%) occurred within clusters, while 14.2% of the variation was attributed to differences among clusters (**Table 3**). The overall FST value was 0.007.

**Table 3:** Genetic diversity statistics and AMOVA based on major genetic clusters inferred from the UPGMA analysis.

Cluster	Number of accessions (%)	mean GD	mean PIC	mean MAF	mean Ho	mean He	mean Fi	F_ST_ among clusters^#^	
								Cluster II	Cluster III
Cluster I	28 (29.8%)	0.22	0.19	0.14	0.23	0.2369	-0.03	-0.004	0.012
Cluster II	23 (24.5%)	0.22	0.18	0.14	0.23	0.2461	-0.07		0.015
Cluster III	40 (42.6%)	0.21	0.18	0.14	0.21	0.2121	-0.01		
AMOVA									
Source of variation		Variation (%)							
Among clusters		14.2%							
Among accessions/within cluster		85.8%							
Overall FST		0.007							
Overall FIS		-0.042							
Overall FIT		-0.034							

GD, Genetic diversity; PIC, Polymorphic index content; MAF, Minor allele frequency; Ho, Observed heterozygosity; He, Expected heterozygosity; Fi, Inbreeding coefficient. ^#^Pair-wise genetic differentiation (F_ST_) among clusters.

**Figure 6:**
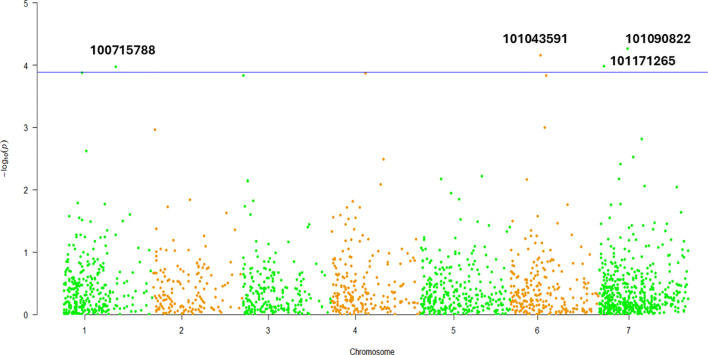
Pseudo Manhattan plot for dry biomass weight in studied *Dactylis glomerata* germplasm.

The mixed linear model (MLM; Q + K) was employed to identify marker-trait associations for dry biomass weight in the *D. glomerata* germplasm. Notably, markers DArT-100715788 and DArT-101043591 were located to Chromosomes 1 and 6, respectively (**Figure 6**; **Table 4**). Additionally, two markers, DArT-101171265 and DArT-101090822, both localized on Chromosome 7, demonstrated significant associations with dry biomass weight, with p-values of 5.44 × 10^-5^ and 1.04 × 10^-4^, respectively (**Table 4**). These findings highlight specific genomic regions linked to dry biomass accumulation.

**Table 4:** Marker trait associations for dry biomass weight in studied *Dactylis glomerata* germplasm.

Trait	Markers	Chromosome	p-Value	MarkerR2
DBW	100715788	1	1.07E-04	0.22593
DBW	101043591	6	6.90E-05	0.2379
DBW	101090822	7	1.04E-04	0.22668
DBW	101171265	7	5.44E-05	0.24445

DBW, dry biomass weight.

## 4 Discussion

Genetic diversity is a critical factor in the study of any species as it directly influences the evolutionary potential and adaptability of populations (Futuyma, 2017). In the context of *D. glomerata*, understanding genetic diversity is essential for characterizing accessions, identifying duplications within germplasm collections, and selecting suitable parental genotypes for breeding programs. Historically, morphological traits have been used to estimate genetic similarity in species, including orchardgrass (Gauthier et al., 1999). However, phenotypic assessments are often unreliable indicators of genetic variation due to environmental influences on phenotype expression. Consequently, molecular approaches offer a more precise method for assessing genetic diversity, providing robust insights into the genetic architecture of *D. glomerata*, which is pivotal for its conservation and improvement.

A total of 2,913 high-quality SNPs was used to assess 91 accessions, resulting in diverse genetic indices across provinces. The highest genetic diversity was observed in accessions from Erzurum (GD = 0.26, He = 0.5328, PIC = 0.21), indicating a rich genetic pool, suggesting that environmental and geographical conditions of Erzurum may favor higher genetic variability. Conversely, lower genetic diversity (GD = 0.15, He < 0.22) in provinces such as Bursa and Muğla suggests greater genetic homogeneity, possibly due to historical bottlenecks, genetic drift, or restricted gene flow in these provinces. These factors may have led to less genetic mixing compared to Erzurum provinces. Conservation and breeding strategies should introduce genetic variation to mitigate risks associated with low diversity, such as reduced adaptability to environmental changes. Comparatively, the observed heterozygosity and expected heterozygosity in these provinces were below the range reported in other studies (Ho = 0.21-0.25, He = 0.44-0.59), which may reflect local adaptation or limited genetic exchange (Last et al., 2013). The genetic distance among accessions ranged from 0.065 (Türkiye3 and Türkiye23) to 0.257 (Kars6 and Türkiye7), with an average of 0.182, reflecting moderate genetic variability across the sampled province. This falls within the range reported for other studies using the AFLP marker on Dactylis (0.0692 to 0.4214; Peng et al., 2006), suggesting that although variability exists, some provinces may have more closely related genetic backgrounds.

The Analysis of Molecular Variance based on provinces indicated that the majority of the genetic variation (95.4%) was attributed to differences among populations while a smaller proportion (4.6%) occurred among individual accessions. This high level of inter-population differentiation is notable and exceeds findings from other studies on Dactylis germplasm using SSR markers, where within-population variation ranged from 63.3% to 74.9% (Xie et al., 2010). The greater variation among populations in this study may be influenced by the geographic isolation of some regions or ecological barriers that limit gene flow, leading to genetic drift and local adaptation.

The highest PIC value based on provincial data was recorded for Erzurum (0.21), which aligns with the findings of Sun et al. (2017), who reported similar PIC in wild *D. glomerata* germplasm using AFLP markers in China. However, our PIC values were lower than those reported in previous studies, such as those of Chtourou-Ghorbel et al. (2024) and Sun et al. (2017), which documented PIC values of 0.69 and 0.25, respectively. Xie et al. (2010) also reported higher PIC values (0.30 and 0.44) than ours in orchardgrass cultivars and breeding lines from North America using SSR markers. The discrepancies among these reports, including the present study, may be attributed to differences in the genetic markers used, sampling strategies, or population structure, which could influence the level of detected genetic diversity. The lower PIC values observed here (0.18-0.21) could be attributed to the bi-allelic nature of SNPs compared to multi-allelic SSR markers, which often capture a broader spectrum of genetic variation (Neuhaus and Horn, 2004). Nonetheless, SNPs offer higher resolution for genome-wide assessments and are suitable for large-scale genetic studies. Our study found greater genetic diversity in *D. glomerata*, with MAF, Ho, and He values surpassing those reported by Hodkinson et al. (2019), who observed a MAF of 0.05, Ho of 0.25, and He of 0.30. This increase may be due to more diverse sampling across regions, better genome coverage using GBS-based SNP markers, and potential historical admixture or outcrossing in our populations. Additionally, natural selection across varied environments could have contributed to maintaining greater genetic variability in our germplasm collection.

The observed genetic distance values and diversity patterns are consistent with previously reported findings across different Dactylis populations, where genetic differentiation often occurs due to regional environmental influences. The average genetic similarity among accessions in other studies ranged widely, from 0.43 to 0.94 (Xie et al., 2010), which supports the observed genetic variability found in this research. However, provinces with lower diversity, such as Bursa and Muğla, may require targeted conservation efforts to enhance genetic variability and reduce the risk of inbreeding. The overall fixation index (FST = 0.143) suggests moderate genetic differentiation, which aligns with findings from Madesis et al. (2014), who reported an FST of 0.186 for Dactylis, indicating that while there is genetic differentiation among populations, gene flow is not entirely restricted. Similar levels of differentiation were observed in other outcrossing grasses, where genetic diversity is primarily maintained within populations (Bolaric et al., 2005; Fjellheim and Rognli, 2005).

The clustering algorithms, UPGMA STRUCTURE, and PCoA were used to assess the genetic differentiation and grouping of the studied germplasm (**Figures 3A, B**, **4**). However, the UPGMA method provided clearer classification by further subdividing the germplasm into distinct subclusters. Therefore, UPGMA was used as the primary clustering method in this study. The UPGMA clustering divided the germplasm into three groups based on their geographical characteristics. In Cluster I, particularly in sub-cluster Ib, all accessions except for Antalya1, Samsun1, and Antalya2 exhibit traits associated with a continental harsh climate.In sub-cluster Ia, the accessions are similar in terms of climate and altitude. Cluster II: sub-cluster IIb contains mostly specimens collected from Turkey without specifying the location. In sub-cluster IIa, except for genotype Sivas3, the other accessions are similar in climate and altitude and close in distance. Cluster III: This cluster contains 40 accessions including the Iranian genotype and except for genotype Muğla2, the accessions are similar in climate and altitude (1000-1350 m).

Furthermore, we also used STRUCTURE for clustering and STRUCTURE also split the studied germplasm into three populations (Population 1, 2 and 3) geographically with few exceptions (**Figure 2A**). Such as Population 1 is the largest population and there are admixture accessions present in this population that are climatically and altitudinally similar moreover, population 2 is climatically similar and close in distance except Erzurum2. Population 3 is climatically and altitudinally similar except for Antalya1, Samsun1, Muğla3, and Antalya2. The PCoA analysis further corroborated the findings of UPGMA, dividing the germplasm into three distinct groups with a clustering pattern largely consistent with UPGMA results (**Figure 4**). This grouping highlights the reliability of the observed genetic differentiation. The grouping reflected similarities based on geographical characteristics and dry biomass weight, a critical trait influencing species adaptability and productivity. The PCoA provided a complementary perspective by visually emphasizing the genetic relationships within and between groups, supporting the robustness of the clustering patterns observed in UPGMA. Together, these analyses highlight the strong genetic structure within the studied germplasm, offering valuable insights for targeted breeding and conservation strategies. We also evaluated the clustering pattern based on dry biomass weight, a critical characteristic of any species of forage (Lutatenekwa et al., 2020; Capstaff and Miller, 2018). The UPGMA cluster analysis based on SNP markers correlated the accessions with their production of biomass. This distinction is particularly pronounced for the high- and low-biomass materials. For instance, all productive accessions were distinctly grouped in cluster II (average dry biomass 128 g/plant), separate from the low-yield accessions in cluster I (100.7 g/plant), whereas a predominant cluster (III) comprised just samples with low biomass (83.8 g/plant) (**Figures 3A, B**). Similar associations between molecular markers-based genetic clustering and biomass productivity have been reported for other forage grass species, such as *Trichloris crinita* (Cavagnaro et al., 2006).

We also measured the genetic diversity indices based on UPGMA clustering. Cluster I showed moderate genetic diversity, with a mean GD of 0.22, PIC of 0.19, and MAF of 0.14. The observed and expected heterozygosities were nearly identical (0.23 and 0.2369, respectively), and the inbreeding coefficient was slightly negative (-0.03), indicates that there is a slight excess of heterozygotes, which may be a result of selection favoring heterozygous individuals, as seen in alfalfa populations where inbreeding proceeds more slowly than expected (Osborn et al., 1997). The intermediate biomass production associated with Cluster I suggests that this genetic makeup may contribute to a balance between genetic variation and trait stability. The slightly higher PIC compared to Cluster II and III implies a relatively higher potential for identifying polymorphisms in this group, possibly due to a more balanced allele distribution, which could be advantageous for breeding programs aiming to improve biomass (Sahu et al., 2020; Thavamanikumar et al., 2011).

Genetic diversity metrics in Cluster II were similar to those of Cluster I, with a GD of 0.22, PIC of 0.18, and MAF of 0.14. However, the expected heterozygosity (0.2461) was marginally higher, and the more negative inbreeding coefficient (Fi = -0.07) suggested a trend toward outbreeding. The higher heterozygosity and potential outbreeding indicate a more diverse genetic pool within Cluster II, which aligns with the higher dry biomass observed in this group. The association between genetic diversity and biomass could be attributed to heterosis (hybrid vigor), where greater genetic variation promotes better adaptation and growth (Fu et al., 2014). The outbreeding trend within this cluster may also enhance the combination of favorable alleles, contributing to increased biomass.

Cluster III is the largest cluster, including 40 accessions, and exhibited slightly lower genetic diversity metrics, with a GD of 0.21, PIC of 0.18, and observed and expected heterozygosity of 0.21 and 0.2121, respectively. The nearly zero inbreeding coefficient (-0.01) suggests balanced allele frequencies and random mating. The low biomass associated with this cluster may be related to the lower genetic diversity observed, potentially limiting the expression of advantageous traits. The reduced diversity could also indicate genetic drift or selection pressure within this group, which may have constrained the range of adaptive genetic variation and resulted in lower trait performance, such as biomass production. The AMOVA results further emphasize the genetic structure observed among the clusters, revealing that 85.8% of the genetic variation occurred within clusters, while only 14.2% was attributed to differences among clusters. This distribution suggests that the majority of genetic diversity is retained within each cluster rather than between them. The low overall FST value of 0.007 indicates minimal genetic differentiation among the clusters, implying a shared genetic background or similar evolutionary forces across the groups. The pairwise FST values between clusters also show limited differentiation, with values ranging from -0.004 between Clusters I and II to 0.015 between Clusters II and III, indicating that the clustering based on genetic similarity rather than geographic origin captures more homogeneity within the groups. Previous studies Sun et al. (2017) and Madesis et al. (2014), reported moderate differentiation in Dactylis populations according to reported FST values of 0.135 and 0.186, respectively. Overall, the results indicate that genetic diversity positively correlates with dry biomass, as evidenced by the higher values in Cluster II, which also had higher heterozygosity. This relationship highlights the importance of maintaining or enhancing genetic diversity in breeding programs to optimize key forage traits like biomass productivity.

This study indicates a novel contribution, as no previous GWAS aimed at DBW in *D. glomerata* has been reported. Significant MTAs were identified, with key markers on Chromosomes 1, 6, and 7, highlighting key genomic regions for biomass accumulation and valuable targets for marker-assisted breeding. Tang et al. (2018) identified 60 QTLs associated with biomass related traits in tetraploid orchardgrass, including traits like plant height, tiller number, and dry weight per plant. Their findings determined significant correlations between these traits and dry weight, providing a way for molecular-assisted breeding. Notably, their identification of QTLs for dry weight on linkage groups overlapping with Chromosome 6 aligns with our findings, highlighting shared genomic regions linked to biomass accumulation, a trait significant for forage yield improvement. Similarities in genetic loci across studies might suggest conserved genomic regions influencing biomass traits across related species, warranting further comparative genomics investigations.

The findings are particularly significant given the context of breeding programs aimed at improving forage yield. The application of GWAS based insights for marker-assisted selection could enhance the efficiency of breeding strategies, as demonstrated by the successful use of such approaches in other forage grasses like perennial buffel grass (Negawo et al., 2024). Future work should expand on these findings by integrating multi-environment trials to validate the stability of these loci under varying environmental conditions. Additionally, functional genomics approaches, such as transcriptomics and CRISPR-based validation, could elucidate the biological mechanisms linking these markers to dry biomass traits. This study paves the way for further *D. glomerata* genetic improvement, which could support sustainable fodder production systems.

## 5 Conclusion

The study provides a comprehensive assessment of the genetic diversity and population structure in *D. glomerata* using GBS-based SNP markers, identifying high variability across accessions and offering valuable insights for conservation and breeding efforts. The high genetic diversity observed in provinces such as Erzurum and Sivas emphasize their potential as genetic reservoirs for breeding programs aimed at improving forage yield, quality, and stress tolerance. In contrast, the lower diversity observed in provinces like Bursa and Muğla underscores the need for targeted conservation strategies to mitigate genetic homogeneity. The association between genetic diversity and biomass production, particularly in Cluster II, highlights the role of heterosis in promoting growth and adaptability. Minimal inbreeding across the accessions further suggests that genetic variability can be effectively maintained through outbreeding strategies. Moreover, GWAS identified significant marker-trait associations for dry biomass weight, a critical agronomic trait, with markers DArT-100715788, DArT-101043591, and DArT-101171265 and DArT-101090822 located on Chromosomes 1, 6, and 7 respectively. These results underline the need for conserving regional diversity while promoting gene flow across populations to optimize *D. glomerata*’s potential in both forage production and environmental resilience. Additionally, the genetic regions identified in this study provide valuable targets for marker-assisted selection, paving the way for advanced breeding programs aimed at improving fodder yield.

## Data Availability

The datasets generated and/or analyzed during the current study are available in the GitHub repository, https://github.com/JosefinaWohlfeiler/Report_DD23-8781_SNP_mapping_1-NAMES.csv. Additional information is presented in the **Supplementary Data** file accompanying this article.
