# Supplementary material for: Genotyping-by-sequencing derived SNP markers reveal genetic diversity and population structure of Dactylis glomerata germplasm
Source: Front Plant Sci. 2025 Feb 6;16:1530585. doi: 10.3389/fpls.2025.1530585 (PMC11840758; doi:10.3389/fpls.2025.1530585)
Supplement: Supplementary file 2 [file Table2.docx]

Supplementary Material

**SUPPLEMENTARY MATERIAL**

**Table S1** Passport data of 91 accessions of *Dactylis glomerata* L.

| **Genotype** | **Species** | **Accessions** | **Country** |
| --- | --- | --- | --- |
| G1 | *Dactylis glomerata* L. | Erzurum1 | Turkey |
| G2 | *Dactylis glomerata* L. | Istanbul2 | Turkey |
| G3 | *Dactylis glomerata* L. | Dogu Yildiz | Turkey |
| G4 | *Dactylis glomerata* L. | Kayseri5 | Turkey |
| G5 | *Dactylis glomerata* L. | Afyon | Turkey |
| G6 | *Dactylis glomerata* L. | Erzincan | Turkey |
| G7 | *Dactylis glomerata* L. | Samsun | Turkey |
| G8 | *Dactylis glomerata* L. | Mugla3 | Turkey |
| G9 | *Dactylis glomerata* L. | Türkiye5 |  |
| G10 | *Dactylis glomerata* L. | Ardahan3 | Turkey |
| G11 | *Dactylis glomerata* L. | Agri | Turkey |
| G12 | *Dactylis glomerata* L. | Türkiye20 | Turkey |
| G13 | *Dactylis glomerata* L. | Ardahan1 | Turkey |
| G14 | *Dactylis glomerata* L. | Çanakkale1 | Turkey |
| G15 | *Dactylis glomerata* L. | Taya | Turkey |
| G16 | *Dactylis glomerata* L. | Elazgi | Turkey |
| G17 | *Dactylis glomerata* L. | Kars4 | Turkey |
| G18 | *Dactylis glomerata* L. | Sivas1 | Turkey |
| G19 | *Dactylis glomerata* L. | Kars6 | Turkey |
| G20 | *Dactylis glomerata* L. | Türkiye3 | Turkey |
| G21 | *Dactylis glomerata* L. | Kars8 | Turkey |
| G22 | *Dactylis glomerata* L. | Türkiye10 | Turkey |
| G23 | *Dactylis glomerata* L. | Kars10 | Turkey |
| G24 | *Dactylis glomerata* L. | Türkiye21 | Turkey |
| G25 | *Dactylis glomerata* L. | Kars1 | Turkey |
| G26 | *Dactylis glomerata* L. | Çanakkale2 | Turkey |
| G27 | *Dactylis glomerata* L. | Malatya | Turkey |
| G28 | *Dactylis glomerata* L. | Kars5 | Turkey |
| G29 | *Dactylis glomerata* L. | Erzurum3 | Turkey |
| G30 | *Dactylis glomerata* L. | Erzurum4 | Turkey |
| G31 | *Dactylis glomerata* L. | Antalya2 | Turkey |
| G32 | *Dactylis glomerata* L. | Türkiye6 | Turkey |
| G33 | *Dactylis glomerata* L. | Türkiye11 | Turkey |
| G34 | *Dactylis glomerata* L. | Kars11 | Turkey |
| G35 | *Dactylis glomerata* L. | Türkiye22 | Turkey |
| G36 | *Dactylis glomerata* L. | Kars2 | Turkey |
| G37 | *Dactylis glomerata* L. | Türkiye26 | Turkey |
| G38 | *Dactylis glomerata* L. | Bingöl1 | Turkey |
| G39 | *Dactylis glomerata* L. | Erzurum2 | Turkey |
| G40 | *Dactylis glomerata* L. | Gümüşhane1 | Turkey |
| G41 | *Dactylis glomerata* L. | Türkiye2 | Turkey |
| G42 | *Dactylis glomerata* L. | Ankara2 | Turkey |
| G43 | *Dactylis glomerata* L. | Türkiye7 | Turkey |
| G44 | *Dactylis glomerata* L. | Türkiye12 | Turkey |
| G45 | *Dactylis glomerata* L. | Türkiye15 | Turkey |
| G46 | *Dactylis glomerata* L. | Türkiye23 | Turkey |
| G47 | *Dactylis glomerata* L. | Ardahan2 | Turkey |
| G48 | *Dactylis glomerata* L. | Türkiye27 | Turkey |
| G49 | *Dactylis glomerata* L. | Bingöl2 | Turkey |
| G50 | *Dactylis glomerata* L. | Erdebill | Iran |
| G51 | *Dactylis glomerata* L. | Kayseri1 | Turkey |
| G52 | *Dactylis glomerata* L. | Kayseri2 | Turkey |
| G53 | *Dactylis glomerata* L. | Bursa | Turkey |
| G54 | *Dactylis glomerata* L. | Türkiye4 | Turkey |
| G55 | *Dactylis glomerata* L. | Ankara3 | Turkey |
| G56 | *Dactylis glomerata* L. | Türkiye13 | Turkey |
| G57 | *Dactylis glomerata* L. | Türkiye16 | Turkey |
| G58 | *Dactylis glomerata* L. | Türkiye24 | Turkey |
| G59 | *Dactylis glomerata* L. | Türkiye1 | Turkey |
| G60 | *Dactylis glomerata* L. | Türkiye28 | Turkey |
| G61 | *Dactylis glomerata* L. | Bingöl3 | Turkey |
| G62 | *Dactylis glomerata* L. | Sivas4 | Turkey |
| G63 | *Dactylis glomerata* L. | Tunceli | Turkey |
| G64 | *Dactylis glomerata* L. | Sivas2 | Turkey |
| G65 | *Dactylis glomerata* L. | Aydın | Turkey |
| G66 | *Dactylis glomerata* L. | Gümüşhane2 | Turkey |
| G67 | *Dactylis glomerata* L. | Kars9 | Turkey |
| G68 | *Dactylis glomerata* L. | Istanbul1 | Turkey |
| G69 | *Dactylis glomerata* L. | Türkiye17 | Turkey |
| G70 | *Dactylis glomerata* L. | Türkiye25 | Turkey |
| G71 | *Dactylis glomerata* L. | Kars3 | Turkey |
| G72 | *Dactylis glomerata* L. | Türkiye29 | Turkey |
| G73 | *Dactylis glomerata* L. | Bingöl4 | Turkey |
| G74 | *Dactylis glomerata* L. | Yozgat | Turkey |
| G75 | *Dactylis glomerata* L. | Kayseri3 | Turkey |
| G76 | *Dactylis glomerata* L. | Mugla1 | Turkey |
| G77 | *Dactylis glomerata* L. | Erzurum5 | Turkey |
| G78 | *Dactylis glomerata* L. | Türkiye8 | Turkey |
| G79 | *Dactylis glomerata* L. | Erzurum6 | Turkey |
| G80 | *Dactylis glomerata* L. | Türkiye18 | Turkey |
| G81 | *Dactylis glomerata* L. | Bayburt | Turkey |
| G82 | *Dactylis glomerata* L. | Antalya1 | Turkey |
| G83 | *Dactylis glomerata* L. | Ayfam | Turkey |
| G84 | *Dactylis glomerata* L. | Kayseri4 | Turkey |
| G85 | *Dactylis glomerata* L. | Ankara1 | Turkey |
| G86 | *Dactylis glomerata* L. | Sivas3 | Turkey |
| G87 | *Dactylis glomerata* L. | Mugla2 | Turkey |
| G88 | *Dactylis glomerata* L. | Kars7 | Turkey |
| G89 | *Dactylis glomerata* L. | Türkiye9 | Turkey |
| G90 | *Dactylis glomerata* L. | Türkiye14 | Turkey |
| G91 | *Dactylis glomerata* L. | Türkiye19 | Turkey |

**
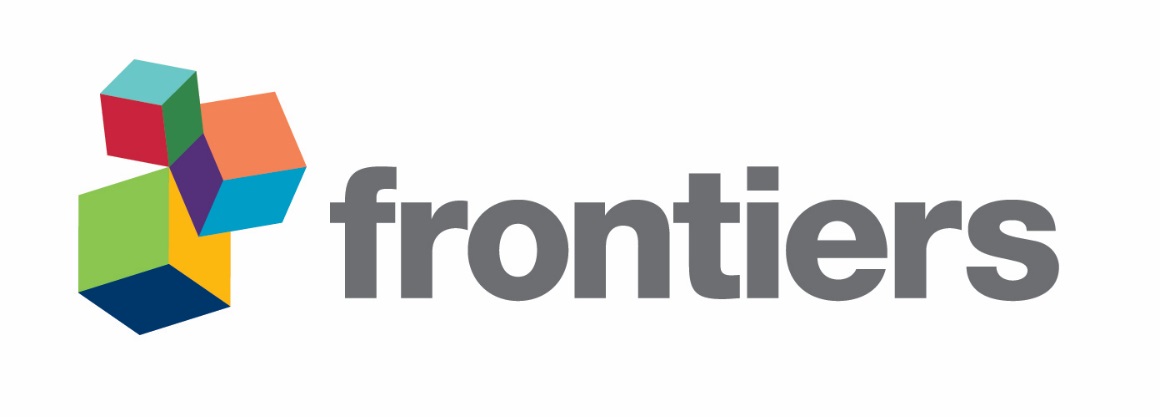
**
